# Timing and Resolution of Bothersome Hot Flashes Following Short Course Oral Gonadotropin-Releasing Hormone Receptor Antagonist Relugolix, and Stereotactic Body Radiotherapy for Localized Prostate Cancer

**DOI:** 10.7759/cureus.104069

**Published:** 2026-02-22

**Authors:** Sarthak Shah, Sukhjeevan Nijhar, Srinivas Sowmiyanarayanan, Omar Anwar, Abigail Pepin, Malika T Danner, Alan L Zwart, Deepak Kumar, Paul D Leger, Nancy A Dawson, Simeng Suy, Sean P Collins

**Affiliations:** 1 Radiation Oncology, Rutgers Cancer Institute of New Jersey, New Brunswick, USA; 2 Radiation Oncology, MedStar Georgetown University Hospital, Washington, USA; 3 Radiation Oncology, Emory College of Arts and Sciences, Atlanta, USA; 4 Radiation Oncology, University of Pennsylvania Abramson Cancer Center, Philadelphia, USA; 5 Biotechnology Research, North Carolina Central University, Durham, USA; 6 Radiation Medicine, University of South Florida Health Morsani College of Medicine, Tampa, USA

**Keywords:** androgen deprivation therapy (adt), gonadotropin-releasing hormone receptor antagonist, hot flashes, prostate cancer, relugolix, sbrt (stereotactic body radiotherapy), stereotactic body radiotherapy (sbrt)

## Abstract

Background

Androgen deprivation therapy (ADT) has been shown to improve cancer control when combined with radiation therapy. Relugolix, an oral gonadotropin-releasing hormone (GnRH) receptor antagonist, suppresses testosterone, causing several hormonally related symptoms that resolve with testosterone recovery. Hot flashes are particularly bothersome. This study sought to evaluate the timeline of hot flashes following a short course of relugolix, and stereotactic body radiotherapy (SBRT) for unfavorable localized prostate cancer.

Methods

IRB approval was obtained for retrospective review of prospectively collected data. Patients were treated at MedStar Georgetown University Hospital per an institutional protocol. Hot flashes were self-reported via question 13a of the Expanded Prostate Index Composite (EPIC)-26 before relugolix initiation, the first day of SBRT treatment, and at subsequent follow-up visits. All patients were treated with the robotic SBRT (Accuray Inc.). Total testosterone levels were measured before SBRT and at each follow-up.

Results

From January 2021 to September 2023, 89 localized prostate cancer patients (63 intermediate, 20 high-risk, and 6 recurrent) at a median age of 72 years (range 49-93) were treated with a short course of relugolix (average 6.25 months, range 3-29 months) and prostate SBRT. The median time to cessation of hot flashes was around nine-months post-SBRT when the median testosterone had recovered in 56% (50) of patients, with a median testosterone of 230 ng/dL. The incidence of burdensome hot flashes (a moderate to big problem) returned to baseline nine-months post-SBRT (0%) with a cumulative incidence of 57% (51). The median EPIC-26 hot flash score of 98.7 declined to 49.0 at one-month post-SBRT and returned to baseline by nine-months post-SBRT. These differences were statistically significant (p< 0.01) and clinically significant (MCID=4.45). Testosterone recovery (>230 ng/dL) occurred in 97% (86) of patients 12-months post-SBRT.

Conclusions

Bothersome hot flashes occur in more than 50% of men treated with short-course relugolix and SBRT. Resolution of hot flashes occurs in the majority of men by nine-months post-SBRT. Hot flash resolution mirrored testosterone recovery at relatively low levels. Reassurance of the temporary nature of hot flashes may reduce patient anxiety and limit associated bother.

## Introduction

The current treatment paradigm for unfavorable localized prostate cancer involves a combination of androgen deprivation therapy (ADT) in combination with radiotherapy [1]. The addition of ADT to radiotherapy improves prostate cancer-specific mortality and distant metastasis rates in men with unfavorable and high-risk prostate cancer [2]. ADT is associated with detriments related to adverse side effects and a reduction in quality of life. In particular, low testosterone levels are associated with hypogonadal symptoms such as fatigue, depression, gynecomastia, weight gain, and hot flashes [3,4].

Hot flashes are one of the most bothersome side effects of ADT. Between 70 to 80% of men will experience hot flashes to variable extents while undergoing ADT [5-7]. Hot flashes are generally benign but may be highly bothersome in select patients [5,8]. Hot flashes are managed with various interventions, but their effectiveness is questionable [9]. Bothersome hot flashes may decrease adherence to ADT recommendations [10]. Hot flashes resolve in the months following discontinuation of ADT, corresponding to testosterone recovery (TR) [5,11].

The timeline to TR following androgen deprivation is variable and dependent on patient and treatment characteristics, including age, race, BMI, and duration of ADT [12-14]. Older and obese men have shorter TR, while black men have faster TR [13,15]. Longer duration of ADT correlates with a slower trajectory to TR [13]. The type of ADT utilized may also influence the timeline of TR [16].

Relugolix is an oral GnRH receptor antagonist that leads to a rapid decrease in testosterone levels to profound castration levels [17-19]. The HERO study, a randomized Phase 3 trial, compared the efficacy of this oral GnRH receptor antagonist with the GnRH agonist leuprolide and demonstrated that relugolix outperformed leuprolide in achieving and maintaining castration [20]. Notably, there were no statistical differences in the incidence of physician or patient-reported hormonal toxicities such as hot flashes [20,21]. Upon discontinuation, patients who were treated with relugolix had a faster and more complete TR to normal levels [20,22]. We previously reported on patients who underwent ADT with lupron [23]. This study sought to understand the onset, severity, and resolution of hot flashes with regards to oral relugolix therapy. Our primary endpoint is the time for hot flash bother to return to baseline as measured by EPIC-26 Q13a.

## Materials and methods

Patient selection

We conducted an IRB-approved, retrospective review of prospectively collected data (IRB 12-1175) of men with prostate cancer. Inclusion criteria included unfavorable, localized prostate cancer treated at Georgetown University Hospital with short-term relugolix and SBRT. Patients were treated per institutional protocol. Risk groups were defined using the D'Amico criteria [24]. Other patient and treatment characteristics, such as age, race, BMI, pre-treatment PSA, T stage, Gleason score, radiation dose, and baseline AUA score, were acquired from the medical records.

Drug treatment

Relugolix was initiated with a loading dose of 360 mg on the first day and continued treatment with a 120 mg dose taken orally once daily at approximately the same time each day as previously described [18]. Men were educated prior to the initiation of relugolix that hormonal symptoms are a known side effect of treatment that would resolve upon drug discontinuation in conjunction with TR. 

Treatment planning and delivery

Prostate SBRT was administered with the CyberKnife robotic radiosurgical system (Accuray, Inc., Sunnyvale, CA) as previously described [18,25,26]. Patients were treated to a prescribed dose of 34-40 Gy to the PTV delivered in 5 fractions over one to two weeks. Treatment beams that directly traversed the testis were blocked, and scatter dose was kept to a minimum [26].

Follow-up and statistical analysis

Before the first SBRT treatment, serum testosterone levels were measured and then monitored during follow-up visits, one-month later, every three-months throughout the first year, and every six-months during the second year. In general, samples were collected in the morning to mitigate the impact of circadian variance [26]. TR was defined as reaching a serum testosterone level of at least 230 ng/dL [27]. Hot flash bother was assessed using the Expanded Prostate Index Composite (EPIC)-26 [28]: Question 13a prior to relugolix initiation, the first day of SBRT, and at each follow-up. The EPIC questionnaire assesses hormonal function, and question 13a specifically assesses bother related to hot flashes [5]. The responses were grouped into three relevant categories (no problem, very small/small problem, and moderate to big problem). Pharmaceutical interventions were not prescribed to reduce hot flashes [9].

To statistically compare changes in EPIC question scores at each time point, the level of responses was assigned a score and transformed onto a 0-100 scale with lower scores reflecting worsening sexual or hormonal symptoms. The minimally important difference (MID=4.45) utilized for the question 13a response was determined by half plus or minus the standard deviation from baseline values [29].

## Results

Eighty-nine patients with unfavorable localized prostate cancer treated with a short-course relugolix and prostate SBRT at Georgetown University Hospital from January 2021 to September 2023 were included in this analysis. Their baseline characteristics are summarized in Table 1. Our patients were ethnically diverse with a median age of 72 years (range 49-93). Thirty-seven percent (33) were non-white patients, and 24% (21) were obese patients. Using the D’Amico risk classification, 63 patients were classified as intermediate, and 20 as high-risk. Patients began relugolix a median of three-months before SBRT (range, 0-11 months) and continued treatment for a median of two-months after SBRT (range, 0-36 months). The mean overall duration of relugolix therapy was 6.25 months (range, 3-29 months; IQR, 5.6-6.8). 

**Table 1:** Patient characteristics and treatment. PSA: Prostate-Specific Antigen; AUA: American Urological Association.

Characteristics	Percentage of Patients (Frequency)
		Age (years): Median 72 (49-93)	N=89
60-69	6.40% (6)
70-79	40% (36)
>80	53% (47)
Race	
White Patients	63% (56)
Black Patients	28% (25)
Other	9.30% (8)
PSA (ng/dL)	Median: 8.2 (± 6.63)
Body Mass Index (kg/m²)	
<18.5	0% (0)
18.5-24.9	29% (27)
25.0-29.9	46% (41)
30.0-34.9	17% (15)
35.0-39.9	6.10% (5)
40.0-44.9	1.20% (1)
Risk Group (D’Amico)	
Intermediate	76% (67)
High	24% (22)
SBRT Dose (Gy)	
35	34% (31)
36.25	66% (68)
AUA Baseline	
0-7 (Mild)	39% (35)
8-19 (Moderate)	45% (40
≥20 (Severe)	16% (14)
Median Prostate Volume (mL)	37.13 (± 23.09)

**Figure 1:**
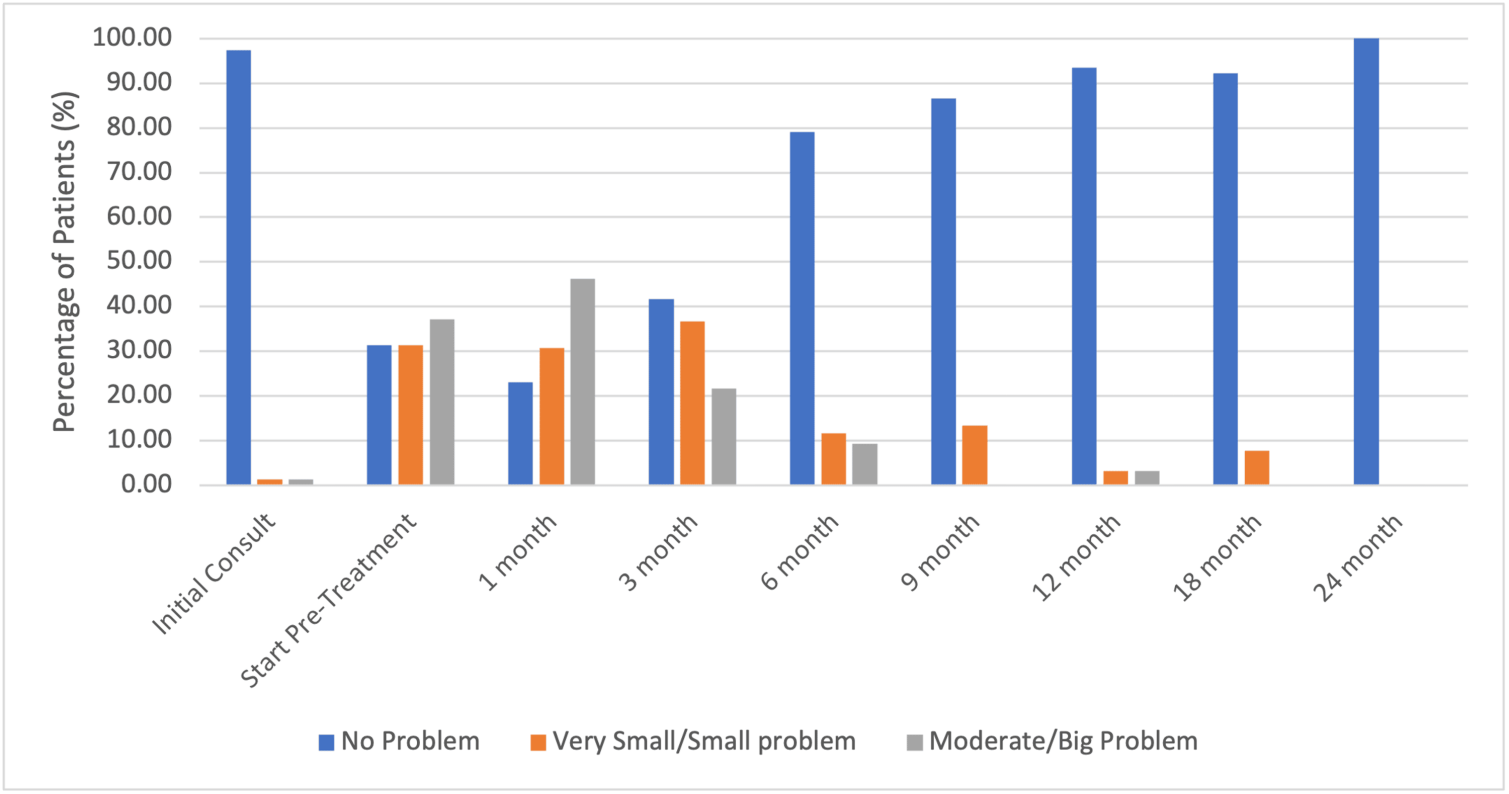
Hot flash bother stratified by bother-type following short course relugolix and stereotactic body radiotherapy (SBRT). Bother of hot flashes across follow-up periods. N=89.

The prevalence of hot flashes before and after SBRT treatment is shown in Table 2 and Figure 1. At the time of the initial consult, 97% (86) of patients reported no problem with hot flashes. Only 1.3% (1) endorsed a very small/small problem, and 1.3% (1) endorsed a moderate/big problem with hot flashes. At one-month post-SBRT, 77% (68) of patients reported hot flashes: 31% (27) described hot flashes as very small or a small problem and 46% (41) as a moderate to big problem. Scores of patient-reported hot flashes decreased significantly following relugolix discontinuation. The median time to cessation of hot flashes was nine-months post-SBRT when the median testosterone had recovered in 56% (50) of patients. The incidence of burdensome hot flashes (a moderate to big problem) returned to baseline at nine-months post-SBRT (0%) with a cumulative incidence of 57% (51).

**Table 2:** Patient-reported problem severity over time (N=89). Hot flashes bother following short-course relugolix and stereotactic body radiotherapy (SBRT) by time point (N=89). Data has been reported as percentages and frequencies.

Time Point	No Problem % (n)	Very Small/Small Problem % (n)	Moderate/Big Problem % (n)
Initial Consult	97.37 % (87)	1.32 % (1)	1.32 % (1)
Start Pre-Treatment	31.4 % (28)	31.4 % (28)	37.21 % (33)
1-month	23.08 % (21)	30.77 % (27)	46.15 % (41)
3-month	41.67 % (37)	36.67 % (33)	21.67 % (19)
6-month	79.07 % (71)	11.63 % (10)	9.3 % (8)
9-month	86.67 % (77)	13.33 % (12)	0 % (0)
12-month	93.55 % (83)	3.23 % (3)	3.23 % (3)
18-month	92.31 % (82)	7.69 % (7)	0 % (0)
24-month	100 % (89)	0 % (0)	0 % (0)

**Figure 2:**
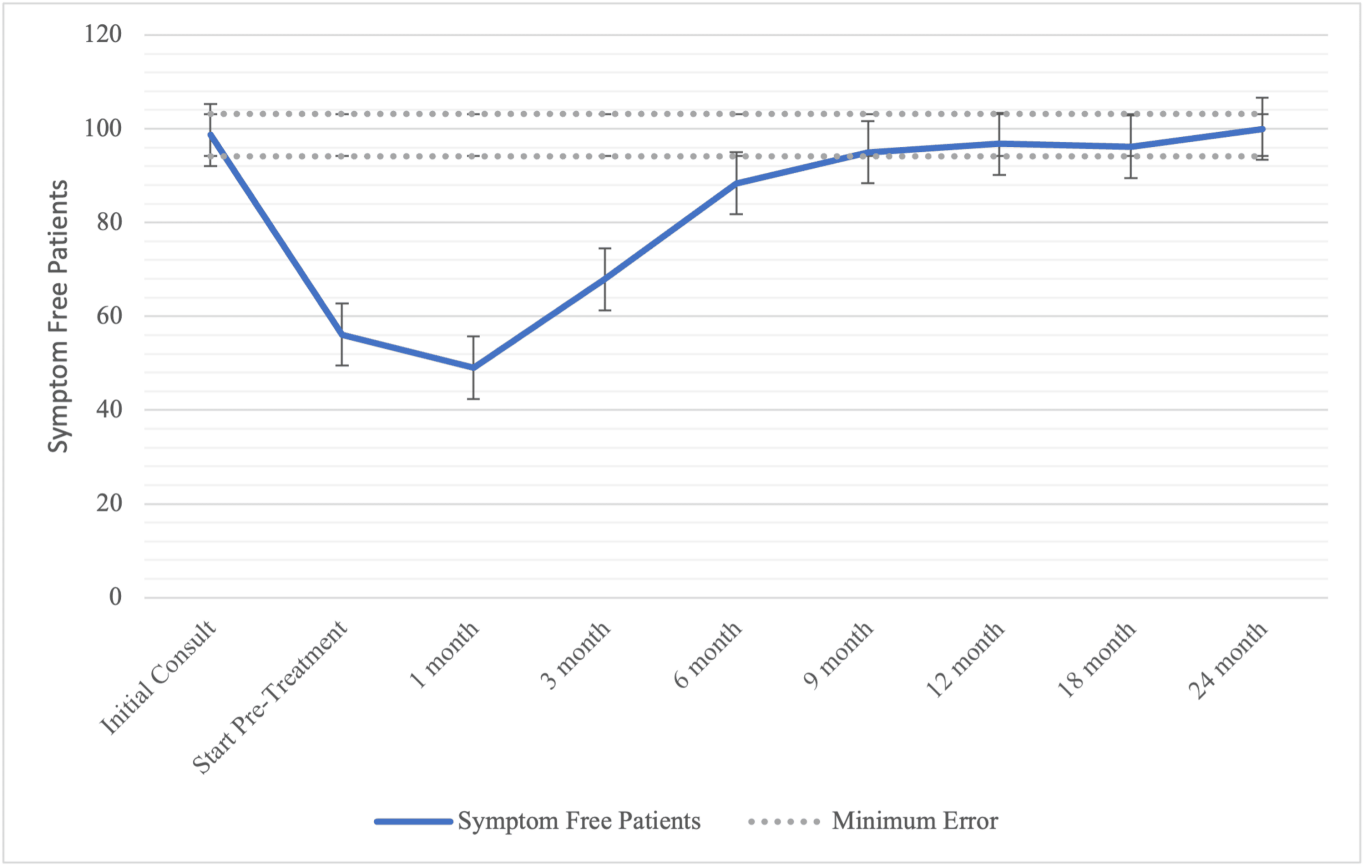
Hot flash reported score over time. Epic 13a transformed scores before and after stereotactic body radiotherapy (SBRT) treatment. Patients were scored from 0 to 100, and the dotted lines indicate the clinically significant range for scores. Hot Flash bother returns to baseline at nine months. N=89.

The hot flash median reported EPIC scores can be seen in Table 3 and Figure 2. Hot flashes were uncommon at baseline, with the median score being 98.7. Hot flash bother increased to 56.1 at the time of radiation start and 49.0 at one-month post-SBRT. Following relugolix discontinuation, scores increased to 68 and 88.4 at three- and six-months-post-SBRT, respectively, and returned to baseline levels by nine-months (95, p=0.58). The hot flash median scores remained stable at this level for the two-year follow-up period, eventually reaching 100 at 24 months (Table 3).

**Table 3:** Hot flash median reported score following short course relugolix and stereotactic body radiotherapy (SBRT) for prostate cancer. N=89

Time of Data Collection	Mean Reported Hot Flash	Standard Deviation
Initial Consult	98.68	8.97
Start Pre-Treatment	56.1	37.08
1-month	49.04	35
3-month	67.92	35.38
6-month	88.37	25.49
9-month	95	13.54
12-month	96.77	13.83
18-month	96.15	13.32
24-month	100	0

**Figure 3:**
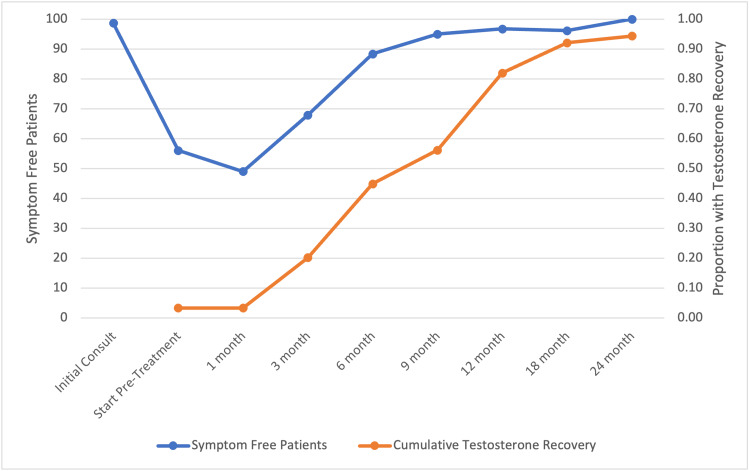
Testosterone and hot flashes over time. Testosterone recovery and Epic 13a scores before and after stereotactic body radiotherapy (SBRT) treatment. Patients were scored from 0 to 100 and testosterone recovery was measured as cumulative incidence across time. N=89.

By six months post-SBRT, testosterone recovered in 45% of patients (Figure 3). There was a strong correlation of 98.6% between testosterone recovery and hot flash score, with both values rising and eventually plateauing as time progressed (R-squared=0.9997) (Figure 3). Testosterone recovery (>230 ng/dL) occurred in 97% (86) of patients 12-months post-SBRT.

## Discussion

Hot flashes are a known complication of relugolix and alternative ADTs [20,21]. While on relugolix, 57% of men reported hot flashes as being a moderate to big problem. This is similar to the 52% we previously reported for men treated with gonadotropin-releasing hormone (GnRH) receptor agonists [5,23]. This may be reflective of the quicker castration seen with patients taking relugolix. The HERO study has previously shown how castration is achieved and maintained faster compared to injectable agents, as well as a quicker TR after completion [21]. Hot flashes may have significant implications for adherence to treatment recommendations [30]. It is important to counsel patients regarding the likely effects of androgen deprivation and discuss the expected recovery with the selected treatment. To our knowledge, this study is the first to report the incidence and severity of hot flashes following relugolix and prostate SBRT. As a novel oral ADT agent, relugolix in combination with prostate SBRT appears to have a favorable recovery regarding bothersome hot flashes.

Herein, we demonstrate a significant correlation between TR and symptomatic hot flashes. Previous studies have demonstrated TR after a short-term GnRH agonist to range from 13 weeks to 2 years [23,31,32]. Similar to what was previously reported by others with short-course relugolix [33], we saw a relatively quick TR following completion of SBRT and discontinuation of relugolix. Fast TR with relugolix was associated with a rapid reduction in hot flash frequency and intensity.

Our results add to previous results demonstrating the limited nature of these side effects [3]. The prevalence of hot flashes peaked at the pre-treatment time point corresponding with the cessation of ADT. We demonstrate that short-term ADT may contribute to hot flash symptoms for up to six-months before recovery to baseline. Bothersome hot flashes were uncommon beyond two years post-SBRT.

A limitation of this study is its retrospective approach. Men may have reported hot flashes only when they became troublesome for themselves or their partners. Additionally, baseline testosterone levels before ADT were not obtained. Hence, the proportion of baseline hypogonadal patients is unknown. Men who had hypogonadism at baseline may have had slower testosterone recovery and more prolonged hot flashes [15,34]. Additionally, relugolix is an oral agent, and adherence is more difficult to monitor when compared to injectable agents. However, testosterone levels served as a marker for adherence and showed that patients remained castrated during the time of treatment, with good TR thereafter.

## Conclusions

Hot flashes are a bothersome self-limiting symptom experienced by a large percentage of men on relugolix. Relugolix allows for castrate levels of testosterone with quick recovery after completion of SBRT, with resolution of bothersome hot flashes in most patients. Hot flashes and testosterone are closely associated with one another, with testosterone recovery coinciding with the resolution of hot flashes. Reassurance of the short duration of hot flashes may assist in reducing patient anxiety. Hot flashes followed the testosterone recovery trend closely, and follow-up monitoring of testosterone levels may allow for guidance to limit the bother of these temporary changes.
